# Impacts of prenatal nutrition on metabolic pathways in beef cattle: an integrative approach using metabolomics and metagenomics

**DOI:** 10.1186/s12864-025-11545-6

**Published:** 2025-04-10

**Authors:** Guilherme Henrique Gebim Polizel, Wellison J. S. Diniz, Aline Silva Mello Cesar, German D. Ramírez-Zamudio, Angela Cánovas, Evandro Fernando Ferreira Dias, Arícia Christofaro Fernandes, Barbara Carolina Teixeira Prati, Édison Furlan, Gabriela do Vale Pombo, Miguel Henrique de Almeida Santana

**Affiliations:** 1 Department of Animal Science, Faculty of Animal Science and Food Engineering, University of São Paulo, Av. Duque de Caxias Norte, 225, Pirassununga, SP 13635-900 Brazil; 2 Department of Animal Sciences, College of Agriculture, Auburn University, Auburn, AL 36849 USA; 3 Department of Food Science and Technology, Luiz de Queiroz College of Agriculture, University of São Paulo, Av. Pádua Dias 11, Piracicaba, SP 13418-900 Brazil; 4 Centre for Genetic Improvement of Livestock, Department of Animal Biosciences, University of Guelph, 50 Stone Road East, Guelph, ON Canada

**Keywords:** Maternal nutrition, Metabolites, Methane, Microbiome, PUFAs, Systems biology, WGCNA

## Abstract

**Background:**

This study assessed the long-term metabolic effects of prenatal nutrition in Nelore bulls through an integrated analysis of metabolome and microbiome data to elucidate the interconnected host-microbe metabolic pathways. To this end, a total of 126 cows were assigned to three supplementation strategies during pregnancy: NP (control)– only mineral supplementation; PP– protein-energy supplementation during the last trimester; and FP– protein-energy supplementation throughout pregnancy. At the end of the finishing phase, blood, fecal, and ruminal fluid samples were collected from 63 male offspring. The plasma underwent targeted metabolomics analysis, and fecal and ruminal fluid samples were used to perform 16 S rRNA gene sequencing. Metabolite and ASV (amplicon sequence variant) co-abundance networks were constructed for each treatment using the weighted gene correlation network analysis (WGCNA) framework. Significant modules (*p* ≤ 0.1) were selected for over-representation analyses to assess the metabolic pathways underlying the metabolome (MetaboAnalyst 6.0) and the microbiome (MicrobiomeProfiler). To explore the metabolome-metagenome interplay, correlation analyses between host metabolome and microbiome were performed. Additionally, a holistic integration of metabolic pathways was performed (MicrobiomeAnalyst 2.0).

**Results:**

A total of one and two metabolite modules associated with the NP and FP were identified, respectively. Regarding fecal microbiome, three, one, and two modules for the NP, PP, and FP were identified, respectively. The rumen microbiome demonstrated two modules correlated with each of the groups under study. Metabolite and microbiome enrichment analyses revealed the main metabolic pathways associated with lipid and protein metabolism, and regulatory mechanisms. The correlation analyses performed between the host metabolome and fecal ASVs revealed 13 and 12 significant correlations for NP and FP, respectively. Regarding the rumen, 16 and 17 significant correlations were found for NP and FP, respectively. The NP holistic analysis was mainly associated with amino acid and methane metabolism. Glycerophospholipid and polyunsaturated fatty acid metabolism were over-represented in the FP group.

**Conclusions:**

Prenatal nutrition significantly affected the plasma metabolome, fecal microbiome, and ruminal fluid microbiome of Nelore bulls, providing insights into key pathways in protein, lipid, and methane metabolism. These findings offer novel discoveries about the molecular mechanisms underlying the effects of prenatal nutrition.

**Clinical trial number:**

Not applicable.

**Supplementary Information:**

The online version contains supplementary material available at 10.1186/s12864-025-11545-6.

## Background

Pregnancy is a physiological status that demands substantial nutrients to meet the fetus’s needs for growth and development and the dam’s maintenance requirements [1]. When divided into three trimesters, bovine pregnancy is characterized by distinct fetal developmental milestones: placentation, organ differentiation, and rapid growth [2, 3]. The extent to which maternal nutritional requirements are met during these periods can significantly impact fetal development [4]. In cow-calf operations in the tropics, the physiological and metabolic conditions of cows, as well as the strategy of more technical production systems, coincide in that the breeding season occurs during the rainy period when forage supply and quality are adequate for reproductive success [5, 6]. However, mid to late pregnancy overlaps with the year’s dry season when forage supply is limited and of low quality [7–9]. Several studies have demonstrated that maternal nutrition during pregnancy has significant phenotypic, molecular, and metabolic effects, with both short and long-term consequences for the offspring [5, 10–12]. To address these challenges and mitigate the potential negative impacts of undernutrition, protein and energy supplementation have been employed as a strategy during critical pregnancy stages in beef cows raised in extensive pasture systems [13].

The influence of maternal nutrition on the offspring gastrointestinal microbiome still needs to be fully elucidated despite well-established evidence that various host factors, including age, breed, genetics, and especially diet, shape the rumen and gut microbiome [14, 15]. While some studies have demonstrated a relationship between the microbiome of cows and their offspring in fecal and ruminal samples [16, 17], the understanding of microbial colonization timing continues to evolve. Traditionally, it was widely accepted that rumen colonization by microorganisms begins at birth [18–20]. However, this notion has been challenged by a recent study on bovine fetuses, which supports the theory of in-utero gastrointestinal tract colonization by bacteria and archaea. The study suggests that colonization may commence as early as the first trimester of gestation [21], significantly altering the perspective on early microbial establishment in ruminants.

Despite the advances in prenatal nutrition research in beef cattle, divergent findings persist in the observed effects on the offspring. Many of these contradictory results are often associated with the variation of nutritional planes applied, including differences in the duration, composition, period and level [4]. Host nutrition broadly impacts the gut and rumen microbiome, health, and many individual microbial taxa. These changes in microbiome composition and diversity are associated with traits such as feed efficiency [22], disease [23], methane emissions [24], and metabolism [25]. To address these complexities and better understand the relationship between host nutrition and microbiota, -OMIC approaches, such as plasma metabolomics [26–28], have been integrated with microbiome analysis. This integration enables more in-depth characterization and insights into the phenotype under study.

Moreover, the lack of holistic studies employing comprehensive and integrative -OMIC approaches has limited our ability to fully understand the causes of these differences. Implementing such studies may be a potential approach to minimize the impact of these differences and advance the knowledge in the fetal programming field. The association among maternal nutrition, offspring metabolome, and microbiome is complex. Key questions remain, such as how prenatal nutrition shapes the metabolome and microbiome, how these changes influence metabolic pathways, and what relationships exist between metabolites and microbial communities. Addressing these questions through integrative approaches will yield valuable insights into the molecular interactions and regulatory mechanisms affected by prenatal nutrition.

The hypothesis of this study is that different maternal nutritional strategies have long-term effects on offspring, impacting metabolic pathways through the integration of both plasma metabolome, and fecal and ruminal microbiome. The objectives were fourfold: (1) To evaluate the impact of prenatal nutritional strategies on the plasma metabolome and microbiome co-abundance networks in the finishing phase; (2) To investigate associations between significant co-abundant metabolites and microbes and their involvement in metabolic pathways; (3) To examine relationships between metabolites and microbial taxa (fecal and ruminal fluid); and (4) To conduct a holistic integration analysis of plasma metabolome and microbiome to assess how prenatal nutrition influences metabolic pathways in beef cattle.

## Methods

### Experimental design

The animals used in this experiment were provided by the Faculty of Animal Science and Food Engineering (FZEA-USP) campus. All animal procedures were approved by FZEA-USP Institutional Animal Care and Use Committee (Protocol #1843241117). Thirty days after the cows’ artificial insemination, which was randomly assigned to semen from four sires, pregnancy was confirmed. The use of semen from multiple sires aimed to provide genetic variability and reduce potential sire-related biases in the study. Based on age, body weight (BW), and body condition score, the dams were divided into three groups of 42 animals each to provide a balanced experimental design. These groups were kept on *Urochloa brizantha* cv. Marandu grazing paddocks that included troughs for water and feed supplementation. The following prenatal treatments were provided to the three groups of cows: Not Programmed (NP (control)), Partial Programming (PP), and Full Programming (FP). The NP cows received only mineral supplements throughout pregnancy (0.03% of their BW per day). The PP cows received protein-energy supplementation during the last trimester of pregnancy equivalent to 0.3% of their BW per day. On the other hand, the FP cows received the same protein-energy supplementation for a longer period, starting from pregnancy confirmation until calving.

The protein-energy supplementation groups (PP and FP) also received mineral supplementation, totaling 0.03% of their BW per day. The composition of the protein-energy supplement already accounted for this mineral addition (as shown in Table 1). During pregnancy, the nutritional values of the grazing paddocks were equivalent across all groups (Table 2) [29]. Detailed information on the pasture conditions, along with the phenotypic and metabolic effects of the treatments (NP, PP, and FP) on dams, was described previously by Schalch Junior et al. [29].

**Table 1:** Composition of prenatal supplement, including the mineral and protein-energy supplement with their respective ingredient and nutrient levels

Ingredients	Mineral Supplement	Protein-Energy Supplement
Corn (%)	35.00	60.00
Soybean meal (%)	-	30.00
Dicalcium phosphate (%)	10.00	-
Urea 45% (%)	-	2.50
Salt (%)	30.00	5.00
Minerthal 160 MD (%)*	25.00	2.50

Nutrients	Mineral Supplement	Protein-Energy Supplement
Total digestible nutrients (%)	26.76	67.55
Crude protein (%)	2.79	24.78
Non-protein nitrogen (%)	-	7.03
Acid detergent fiber (%)	1.25	4.76
Neutral detergent fiber (%)	4.29	11.24
Fat (%)	1.26	2.61
Calcium (g/kg)	74.11	6.20
Phosphorus (g/kg)	59.38	7.24

*Mineral premix composition (Minerthal company): Calcium = 8.6 g/kg; Cobalt = 6.4 mg/kg; Copper = 108 mg/kg; Sulfur = 2.4 g/kg; Fluorine = 64 mg/kg; Phosphorus = 6.4 g/kg; Iodine = 5.4 mg/kg; Manganese = 108 mg/kg; Selenium = 3.2 mg/kg; Zinc = 324 mg/kg; Sodium monensin = 160 mg/kg [90]

**Table 2:** Nutrient composition of the pastures consumed by cows in the different groups

Pasture nutrients	NP	PP	FP
CP % (crude protein)	7.38 ± 1.72	7.82 ± 2.28	7.40 ± 2.30
TDN % (total digestible nutrients)	63.1 ± 1.45	64.1 ± 2.33	61.4 ± 2.12
NDF % (neutral detergent fiber)	59.0 ± 3.67	61.4 ± 5.05	58.4 ± 4.11
Ca % (calcium)	0.38 ± 0.11	0.35 ± 0.05	0.39 ± 0.08
P % (phosphorus)	0.19 ± 0.03	0.19 ± 0.03	0.17 ± 0.03

(NP, PP, and FP), with values presented as mean ± standard deviation [29]

After calving, protein-energy supplementation was stopped. All offspring, regardless of their prenatal nutritional treatment, followed the same health procedures and dietary regimens until weaning at 240 ± 28 days. During this period, cows had the same mineral supplementation (0.03% of BW) as during the pregnancy phase and were kept on an extensive grazing system of *Urochloa brizantha* cv. Marandu paddocks.

### Rearing and finishing phase

Post-weaning, animals were separated by sex, regardless of their prior treatment, and reared until the end of the developmental phase at 570 ± 28 days under the same nutritional management. During this phase, young bulls received two types of supplements: an energy supplement (TDN = 67.55%; CP = 24.78%; fat = 2.61%; 0.3% of BW) during the dry season (winter), and a protein supplement (TDN = 53.15%; CP = 30.03%; fat = 1.65%; 0.1% of BW) during the wet season (summer). From calving until 570 ± 28 days of age, the young bulls grazed on *Urochloa brizantha* cv. Marandu pastures with free access to water.

The finishing phase for the 63 bulls started at 570 ± 28 days of age and ended with slaughter at 676 ± 28 days. During this phase, the bulls were fed three different diets: an initial adaptation diet for 15 days, followed by a second diet for 35 days, and a final diet for 56 days. These diets were formulated to gradually adjust the bulls’ nutrition throughout the finishing phase. Additional information about the finishing diet was reported elsewhere [30].

After completing the finishing phase, the bulls were slaughtered at the FZEA/USP school slaughterhouse, which is located approximately 500 m from the feedlot facilities. The average weights and ages at slaughter were as follows: NP = 591.2 ± 40.05 kg (678 ± 29 days), PP = 602.6 ± 49.65 kg (676 ± 29 days), and 597.4 ± 51.06 kg (675 ± 28 days). The slaughter process and subsequent carcass processing were carried out in accordance with the guidelines set by the Ministry of Agriculture, Livestock, and Supply of Brazil (MAPA), as specified in Normative Instruction No. 9 of 2004. Detailed phenotypic information about slaughter, gastrointestinal and meat parameters are available in [30, 31].

### Plasma, fecal and ruminal sample collection

Blood and fecal samples from 63 Nelore bulls were collected at 660 ± 28 days of age, 16 days prior to slaughter. The rumen fluid samples were collected in the slaughterhouse, within 15 min after slaughter (676 ± 28 days), during the processing of the animals. For this study, 5 experimental units were randomly selected from each treatment group (Plasma, *n* = 15; Feces, *n* = 15; Ruminal fluid, *n* = 15), with the same animals included in all analyses.

Blood samples were aseptically collected from the jugular vein of each animal into EDTA-coated tubes (BD Vacutainer, São Paulo, Brazil) to prevent coagulation. Immediately after collection, the tubes were placed on ice to maintain sample integrity and minimize metabolic activity. All blood samples were processed within one-hour post-collection to ensure consistency and reliability of downstream analyses. To separate plasma, the blood samples were centrifuged at 3,000×g for 10 min at 4 °C using a refrigerated centrifuge. This step allowed for the effective separation of plasma from cellular components. Following centrifugation, the plasma supernatants were carefully aspirated to avoid contamination with the buffy coat or red blood cells and transferred to pre-labeled, sterile microcentrifuge tubes. The plasma aliquots were then immediately stored at -80 °C to preserve their biochemical properties and prevent degradation until further analysis.

Stringent protocols were used to obtain fecal and ruminal fluid samples for metagenomics analysis, ensuring sample integrity and minimizing contamination. Fecal samples were collected directly from the rectum of each animal to retrieve fresh material. Disposable gloves were used throughout collection to minimize cross-contamination, and sterile, pre-labeled microtubes were used to collect around 10–20 g of feces from each animal. Following the slaughtering and evisceration, ruminal fluid samples were filtered with 4 layers of sterilized cheesecloth and placed in conical tubes (20 mL per animal). The tubes (feces and ruminal fluid) were initially kept in ice packs and later stored in an ultrafreezer at -80 °C until DNA extraction.

### Plasma targeted metabolomics

The AbsoluteIDQ^®^ p180 Kit (Biocrates Life Sciences, Innsbruck, Austria) was utilized to conduct targeted metabolomics analyses on plasma samples. This kit measures 188 metabolites (21 amino acids, 21 biogenic amines, 40 acylcarnitines (Cx), 14 lysophosphatidylcholines (lysoPC), 76 phosphatidylcholines (PC), 15 sphingolipids (SMx), and one monosaccharide). The analyses were conducted by Apex Science Company (Campinas, São Paulo, Brazil) using the SCIEX 4000 series^®^ system. Amino acids and biogenic amines were quantified using high-performance liquid chromatography tandem mass spectrometry (HPLC-MS/MS) with electrospray ionization. Lysophosphatidylcholines, phosphatidylcholines, acylcarnitines, and hexose were assessed via flow injection analysis-tandem mass spectrometry (FIA-MS/MS). The quantification and identification of metabolites were based on Biocrates MetIDQ™ software and its proprietary library, which contains prevalidated reference standards and optimized methods tailored for the p180 Kit. The data were processed using MetIDQ^®^ software, and metabolite concentrations were determined using isotopically labeled internal standards, ensuring high precision and reproducibility. Biocrates employs rigorous quality control measures, including experimental methods to determine metabolite-specific limits of detection (LOD) for each analyte. Three distinct levels of quality control samples were included on the analysis plate as described in the user manual. These controls consist of lyophilized human plasma samples spiked with low, medium, and high concentrations of target metabolites. The performance of these quality control samples was assessed using the MetVAL module of the MetIDQ^®^ software, and the results were displayed in both the MetVAL module and the MetSTAT results table. The inclusion of these quality control measures ensures that the analytical process adheres to the highest standards of accuracy and reliability.

### Metabolomics data processing

The identified metabolites were filtered based on the LOD established by the Biocrates company. Initially, values exceeding the LOD (observed in nine of the 188 metabolites, representing 4.78%) were treated as missing data. Metabolites with more than 70% of their values falling below or above the LOD threshold were subsequently removed from the dataset (six metabolites were removed; 3.19%) ensuring that at least 5 animals had values within the LOD range. Compounds with uniform values across the samples were also excluded (five metabolites were removed; 2.66%), resulting in a final dataset of 177 metabolites. For the metabolites retained after filtering, values below the LOD were replaced with the minimum detected value, while values above the LOD were replaced with the maximum detected value for each respective variable. This adjustment was applied to three metabolites, accounting for 1.69% of the dataset. This approach ensured that the dataset was suitable for subsequent analyses. The dataset was autoscaled using the “scale” function in the R statistical environment v.4.4.0 to meet normalization requirements and prepare it for further analyses. The raw metabolomics dataset is available on the Additional file 1.

### DNA extraction, PCR amplification and 16 S rRNA sequencing from fecal and ruminal fluid samples

The Macherey Nagel NucleoSpin Tissue^®^ commercial kit was used to extract the DNA, and 16 S rRNA gene sequencing was performed on the MiSeq platform (2 × 250 bp; Illumina, San Diego, CA, USA) using primers for the V3 to V4 region, as recommended by the manufacturer. The methodology involved two PCR steps. The first step amplified the targeted region of the 16 S rRNA gene from the template DNA, including the Illumina adapter sequences. The PCR products were then purified using AMPure XP beads, and fragment sizes were evaluated via agarose gel electrophoresis. In the second step, barcodes from the Nextera XT kit were attached. This was followed by additional PCR purification and library validation steps. The libraries were quantified to ensure uniform sample pooling into a single library.

The Illumina forward overhang adapter sequence [5ʹ-TCGTCGGCAGCGTCAGATGTGTATAAGAGACAG-(locus-specific sequence)-3´] and reverse overhang adapter sequence [5ʹ-GTCTCGTGGGCTCGGAGATGTGTATAAGAGACAG‐(locus-specific sequence)-3´] were completed with the locus-specific sequence using S-D-Bact-0341-b-S-17 (5′-CCTACGGGNGGCWGCAG-3′) as forward primer, and S-D-Bact-0785-a-A-21 (5′-GACTACHVGGGTATCTAATCC-3´) as reverse primer [32].

A heterogeneous control, the phi-X phage, was combined with the pool of amplicons. Finally, the libraries and phi-X were denatured to allow sequencing.

### Metagenomics data processing

The processing of amplicon metagenomic data was carried out on the statistical environment R v.4.4.0. The DADA2 v.1.32.0 workflow [33] was used to infer the Amplicon Sequence Variants (ASVs) and for the taxonomic assignments. Briefly, we first filtered and trimmed the raw sequencing reads to remove poor-quality bases (quality score ≥ 30) and adapter contamination. Identical reads were then combined (dereplication). The reads were then denoised, merged, and filtered to remove artifacts related to PCR and PhiX-related chimeras. The ASVs were quantified and taxonomically annotated using the SILVA database of non-redundant sequences (version v.138.1, nr99) [34]. The data were structured in objects including the ASVs quantifications, the taxonomy annotations and the sample treatments groups (NP, PP and FP) through the phyloseq package v.1.48.0 [35]. The phyloseq was used to remove phyla with just one feature and to filter for prevalence (≥ 20%; at least three samples with counts). After all filtering steps, the fecal (Additional file 2) and ruminal fluid (Additional file 3) dataset resulted in 258 and 302 microbial taxa (genus level), respectively. Fecal and ruminal fluid microbiome taxonomy table can be accessed in Additional files 4 and 5, respectively. The final datasets were then log2 transformed for further analyses. Results regarding taxa abundance and diversity can be found in [30].

### WGCNA analysis

The Weighted Gene Co-expression Network Analysis (WGCNA) R-package v. 1.72-5 [36] was used to explore the co-expression pattern of metabolites and ASVs in response to the prenatal nutrition effects. In addition, the WGCNA approach was used to reduce the data dimensionality, and the results were used for further analyses.

The first stage of the WGCNA analysis required turning our nutritional treatment groups (NP, PP, and FP) into binary variables using dummy transformation to match the WGCNA requirements. An adjacency matrix was generated using the WGCNA framework, which estimated the Spearman’s correlation coefficients between metabolite pairs and ASVs pairs datasets separately. To create co-abundance networks following the scale-free assumptions (R^2^ ≥ 0.80) [37], soft thresholds were established for the plasma metabolome (power = 12, R^2^ = 0.92), fecal microbiome (power = 11, R^2^ = 0.85), and rumen fluid microbiome (power = 18, R^2^ = 0.93).

Following this, the adjacency matrix was converted into a topological overlap matrix. The cluster analysis was then carried out to detect modules, ensuring a minimum of five components per module for both -OMICs (metabolomics and metagenomics) [38, 39]. Modules having a correlation value (r) of 0.75 or higher were merged within each dataset. Using hierarchical clustering (Additional file 6), metabolites or microbial taxa with a similar abundance pattern (individually in each -OMIC dataset) across samples were placed into the same module and randomly designated by color. The modules were then summarized based on the eigengene concept, and the module eigengene values were correlated (Spearman’s correlation) with the groups (NP, PP, and FP). Modules were considered significant when the p-value was ≤ 0.1, according to WGCNA manual guidelines. Heatmaps were then created to show the correlations (with corresponding p-values) between the groups and the metabolite and microbial taxa modules.

### Functional enrichment analysis

Functional enrichment analyses to uncover metabolic pathways underlying each significant module were conducted for both ASVs and metabolites. To perform ASVs functional enrichment analysis, we first used the EMBOSS Transeq tool (https://www.ebi.ac.uk/jdispatcher/st/emboss_transeq) to translate the nucleotide FASTA sequences into amino acid FASTA sequences. These sequences were then annotated using the database genus_prokaryotes + viruses of the GhostKOALA [40]. This web service maps genes to KOs, which represent groups of orthologous genes associated with specific molecular functions. By analyzing the KO content, organisms and samples can be clustered, revealing distinct groups with unique characteristics. These groups are identified through various data mining techniques and primarily, though not exclusively, reflect differences in metabolic potential [41]. Ultimately, these insights facilitate the formulation of targeted hypotheses about the physiological traits of individual ASVs and the broader community within specific samples and the prenatal nutritional treatments applied. Over representation analysis was carried out using the MicrobiomeProfiler v.1.10.0 R package to identify the metabolic pathways using KEGG (Kyoto Encyclopedia of Genes and Genomes) database involved in each significant module generated by the WGCNA analysis. Metabolic pathways with FDR (False Discovery Rate) ≤ 0.1 were considered significant.

Metabolomic data enrichment analysis was conducted by inputting the list of metabolites identified in each significant module into the MetaboAnalyst v.6.0 platform [42] The “Over Representation Analysis” function was used to identify enriched metabolic pathways. This approach evaluates whether certain metabolites are overrepresented within specific pathways, providing insights into the biological relevance of the metabolite modules. Pathway analysis was based on the KEGG database, ensuring comprehensive coverage of known metabolic pathways. Additionally, chemical structures (sub-classes) of the metabolites within each module were examined to further characterize the modules. Significance was determined using an adjusted p-value threshold of ≤ 0.1.

### Metagenomics-metabolomics data integration

Two approaches were used to integrate the metagenomics and metabolomics data: (1) Spearman’s correlation analysis between the plasma metabolites and ASVs for each treatment, and (2) integration of all significant components from each treatment, regardless the sample tissue, to fully understand the impact of prenatal nutrition on metabolic pathways. Our inputs comprised all ASVs and metabolites from significantly associated modules. It is worth noting that the PP treatment was not included in the integration analyses because it did not show any significant modules in the plasma WGCNA analysis.

The Spearman’s correlation analysis was used to identify host metabolites and ASVs significantly correlated, and consequently associated with the prenatal nutritional treatments (NP and FP). This method was selected due to its robustness to non-normal distributions and non-linear relationships, particularly relevant as metabolites were autoscaled and ASVs log2-transformed. Significant correlations were visualized as heatmaps, highlighting associations influenced by prenatal nutrition.

**Fig. 1:**
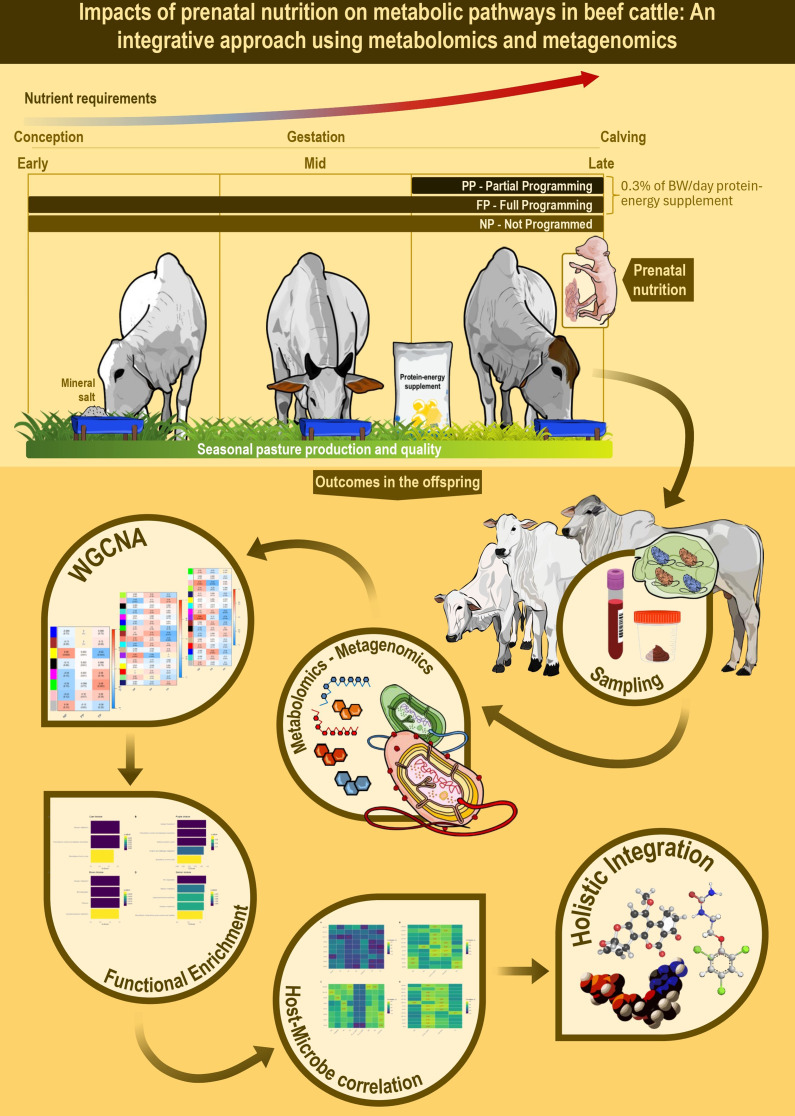
Experimental design and workflow illustrating sample collection, treatments, and analyses performed

Additionally, the NP and FP treatments were also individually analyzed (regardless of the -OMIC technology or approach or tissue type used) to identify the differential metabolic pathways affected by prenatal nutrition. The MicrobiomeAnalyst v.2.0 [43] was employed to obtain functional insights, carrying out an integrated over representation analysis across the whole metabolic map involving the three tissues simultaneously (plasma metabolome, fecal and ruminal microbiome). For this analysis, significant components identified in each prenatal treatment (NP and FP) were imputed. Metabolic pathways with FDR ≤ 0.1 were considered significant, and common metabolic pathways between the treatments were removed. Figure 1 illustrates the workflow of the analyses conducted, along with the experimental design, providing a comprehensive overview of each step performed.

## Results

### WGCNA analysis

**Fig. 2:**
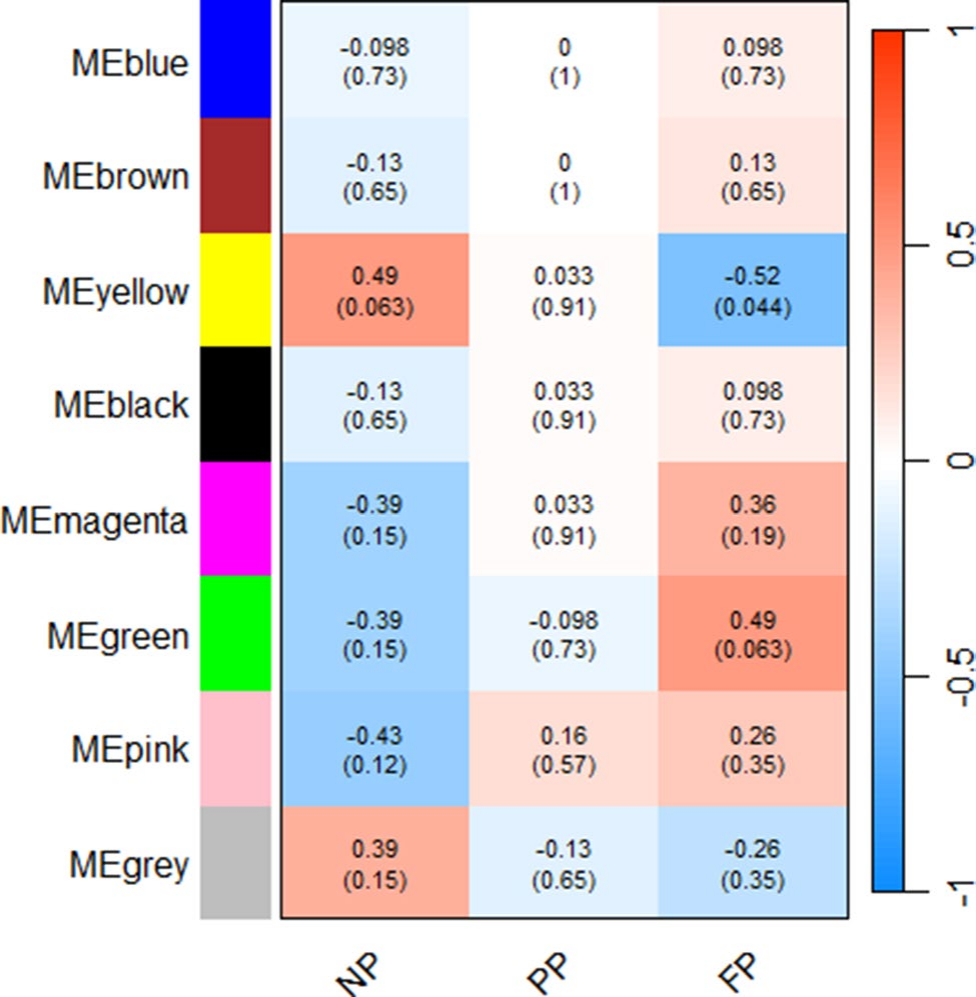
Plasma metabolome module–treatment correlation heatmap. Each row corresponds to a metabolite module, and each column corresponds to a prenatal nutritional treatment group (NP, PP, and FP). Each cell contains the corresponding correlation and p-value. The table is colour-coded by correlation, according to the colour legend

The metabolome co-abundance analysis identified one significant module for the NP group (yellow), with a correlation coefficient of 0.49, and two significant modules for the FP group (yellow and green), with correlation coefficients of -0.52 (yellow) and 0.49 (green). No significant modules were found for the PP group. Notably, while the yellow module was shared between the NP and FP groups, it exhibited opposite correlation signs, suggesting different metabolic behaviors between these groups (Fig. 2).

**Fig. 3:**
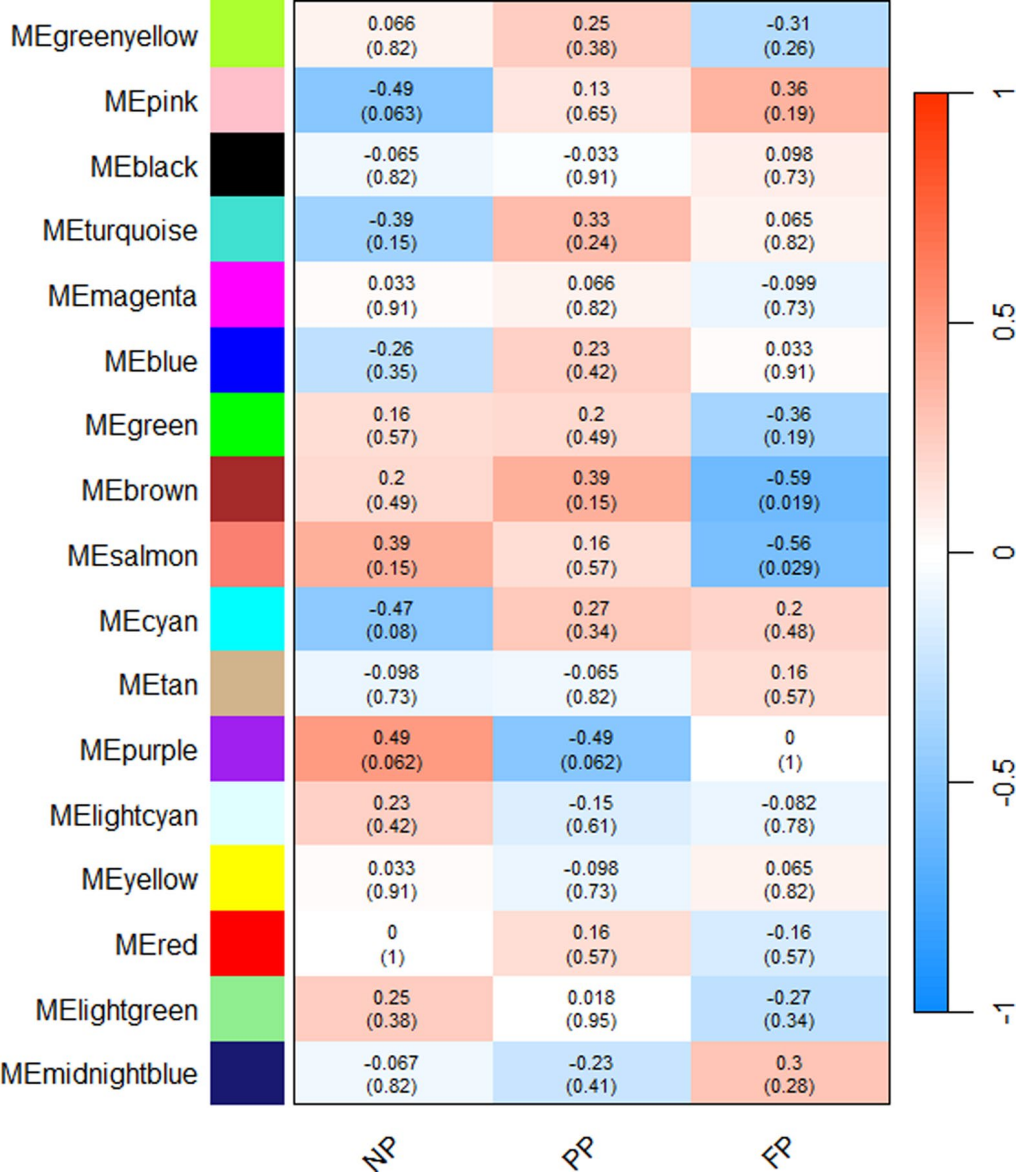
Fecal microbiome module–treatment correlations heatmap. Each row corresponds to an ASV module, and each column corresponds to a prenatal nutritional treatment group (NP, PP, and FP). Each cell contains the corresponding correlation and p-value. The table is colour-coded by correlation, according to the colour legend

The significant correlations (*p* ≤ 0.1) regarding fecal microbiome ranged from|0.47| to|0.49| in the NP group modules, and|0.56| to|0.59| in the FP group modules. In the PP group, only one significant module was correlated with the fecal microbiome (purple module [*r* = -0.49]). As demonstrated in the Fig. 3, we also identified shared modules between the groups in the fecal microbiome. The NP and PP groups shared the purple module, showing opposite correlation (0.49 and − 0.49, respectively). In addition, the NP group showed two more significant modules (pink [*r* = -0.49] and cyan [*r* = -0.47]). The FP group showed significant negative correlations with the brown (*r* = -0.59) and salmon (*r* = -0.56) modules.

Regarding the ruminal fluid microbiome, the NP group was correlated (*p* ≤ 0.1) with pink and purple modules (*r* = 0.52; *r* = 0.69, respectively). The pink module was likewise shared with PP group (*r* = -0.59), while the purple module was shared with the FP group (*r* = -0.54), both indicating opposite correlations between the groups. Thus, the metabolic pathways associated with each module act differently among the prenatal groups. Furthermore, the PP group was exclusively correlated with the blue module (*r* = 0.46), and the FP group uniquely correlated with the magenta module (*r* = -0.53). Table 3 presents all the components of each significant module identified in the WGCNA analyses.

**Table 3:** Components of the significant modules from WGCNA analysis. The table lists the corresponding -OMIC dataset, tissue type, module color, and the components associated with each significant module

-OMICS	Tissue	Modules	Components
Metabolome	Plasma	Yellow	Ala; Arg; Asn; Asp; Gln; Glu; Gly; His; Ile; Leu; Lys; Met; Orn; Phe; Pro; Ser; Thr; Tyr; Val; Ac.Orn; Carnosine; t4.OH.Pro; C3-DC.C4(OH)
Metabolome	Plasma	Green	Alpha.aaa; ADMA; Creatinine; Serotonin; C10.1; C10.2; C12.1; C14.1; lysoPC.a.C16.1; lysoPC.a.C17.0; lysoPC.a.C18.1; lysoPC.a.C20.3; lysoPC.a.C24.0; lysoPC.a.C26.0; lysoPC.a.C28.0; lysoPC.a.C28.1; PC.aa.C26.0; PC.aa.C30.0; PC.aa.C32.0; PC.aa.C34.1; PC.aa.C36.0; PC.aa.C36.1; PC.aa.C36.3; PC.aa.C36.5; PC.aa.C36.6; PC.aa.C38.3; PC.aa.C38.4; PC.aa.C38.5; PC.aa.C40.1; PC.aa.C40.2; PC.aa.C40.3; PC.aa.C40.4; PC.aa.C40.5; PC.aa.C42.2; PC.aa.C42.4; PC.ae.C30.0; PC.ae.C30.1; PC.ae.C30.2; PC.ae.C32.2; PC.ae.C34.0; PC.ae.C34.3; PC.ae.C36.0; PC.ae.C36.1; PC.ae.C38.0; PC.ae.C38.1; PC.ae.C38.2; PC.ae.C38.3; PC.ae.C38.4; PC.ae.C40.1; PC.ae.C40.2; PC.ae.C40.3; PC.ae.C40.4; PC.ae.C40.5; PC.ae.C42.0; PC.ae.C42.1; PC.ae.C42.2; PC.ae.C42.3; PC.ae.C42.4; PC.ae.C42.5; SM.OH.C22.2; SM.OH.C24.1; SM.C16.0; SM.C16.1; SM.C24.0; SM.C26.0
Metagenome	Feces	Pink	ASV9; ASV16; ASV17; ASV19; ASV29; ASV38; ASV60; ASV65; ASV82; ASV104; ASV111; ASV112; ASV119; ASV180; ASV463
Metagenome	Feces	Cyan	ASV76; ASV169; ASV174; ASV189; ASV217; ASV268; ASV270; ASV552; ASV567
Metagenome	Feces	Purple	ASV69; ASV81; ASV89; ASV113; ASV117; ASV181; ASV234; ASV245; ASV309; ASV330; ASV437
Metagenome	Feces	Brown	ASV7; ASV8; ASV15; ASV21; ASV24; ASV25; ASV26; ASV28; ASV30; ASV31; ASV32; ASV33; ASV34; ASV37; ASV40; ASV46; ASV50; ASV54; ASV58; ASV72; ASV73; ASV86; ASV92; ASV95; ASV328
Metagenome	Feces	Salmon	ASV97; ASV98; ASV116; ASV149; ASV182; ASV205; ASV213; ASV238; ASV275; ASV751
Metagenome	Rumen fluid	Pink	ASV39; ASV51; ASV53; ASV63; ASV65; ASV68; ASV87; ASV95; ASV109; ASV131; ASV142; ASV215; ASV220; ASV290; ASV387
Metagenome	Rumen fluid	Purple	ASV12; ASV34; ASV57; ASV76; ASV100; ASV133; ASV186; ASV198; ASV231; ASV587; ASV648; ASV861; ASV1035
Metagenome	Rumen fluid	Blue	ASV21; ASV78; ASV89; ASV91; ASV110; ASV158; ASV192; ASV197; ASV200; ASV230; ASV233; ASV245; ASV276; ASV347; ASV402; ASV458; ASV520; ASV547; ASV1042; ASV1139
Metagenome	Rumen fluid	Magenta	ASV9; ASV27; ASV30; ASV45; ASV67; ASV83; ASV85; ASV96; ASV101; ASV116; ASV268; ASV309; ASV322; ASV443; ASV1045

**Fig. 4:**
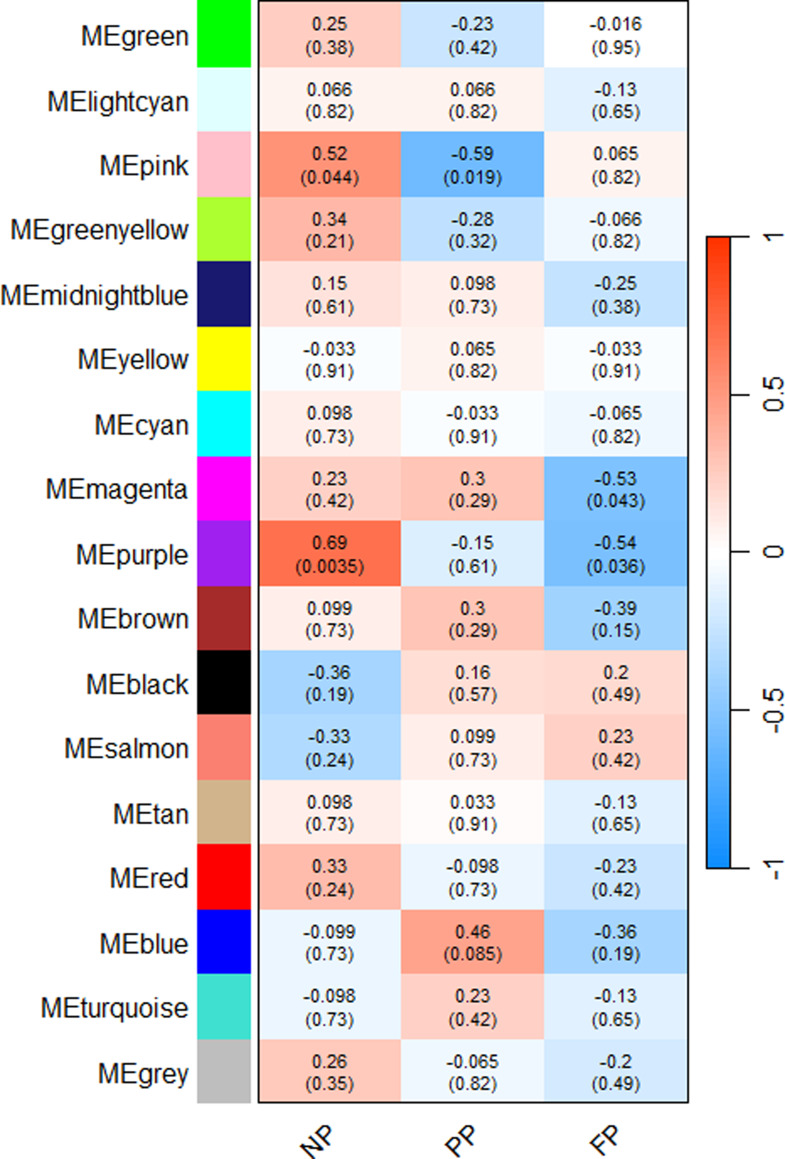
Rumen fluid microbiome module–treatment correlations heatmap. Each row corresponds to an ASV module, and each column corresponds to a prenatal nutritional treatment group (NP, PP, and FP). Each cell contains the corresponding correlation and p-value. The table is colour-coded by correlation, according to the colour legend

### Metabolome functional enrichment analysis

Based on the metabolome functional enrichment analysis of each significant module, we found several important metabolic pathways and sub-classes (adjusted p-value ≤ 0.1) related to the prenatal treatments.

The yellow module showed that all metabolic pathways were associated with amino acid metabolic processes (arginine biosynthesis [*p* = 9.13e-07]; valine, leucine and isoleucine biosynthesis [*p* = 8.47e-05]; alanine, aspartate and glutamate metabolism [*p* = 7.7e-04]; histidine metabolism [*p* = 0.001]; arginine and proline metabolism [*p* = 0.001]; glyoxylate and dicarboxylate metabolism [*p* = 0.010]; phenylalanine, tyrosine and tryptophan biosynthesis [*p* = 0.013]; nitrogen metabolism [*p* = 0.025]; beta-alanine metabolism [*p* = 0.025]; phenylalanine metabolism [*p* = 0.041]; glutathione metabolism [*p* = 0.048]; and glycine, serine and threonine metabolism [*p* = 0.069]).

**Fig. 5:**
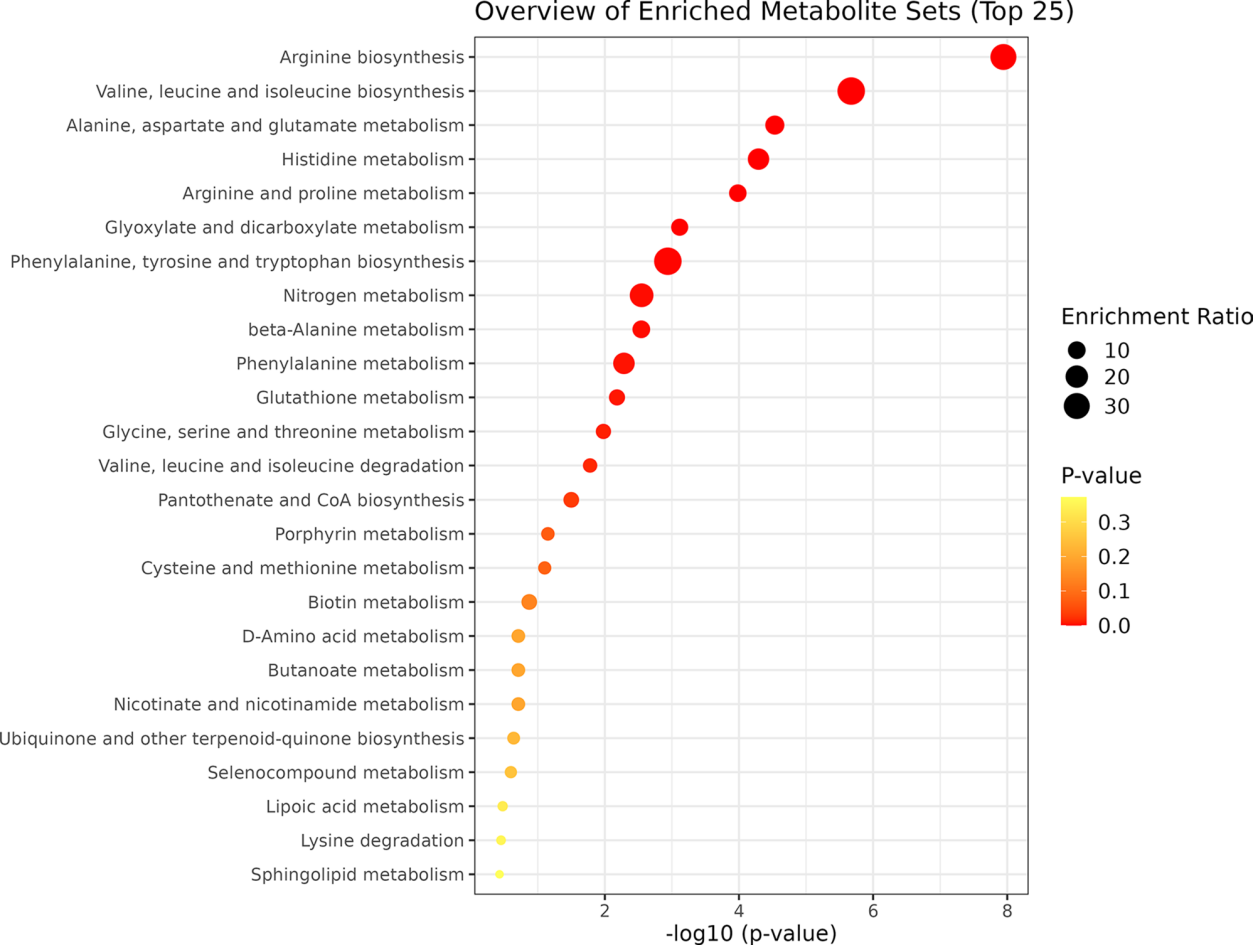
Bubble plot illustrating the over-representation analysis of metabolites associated with the yellow module from plasma WGCNA analysis

**Fig. 6:**
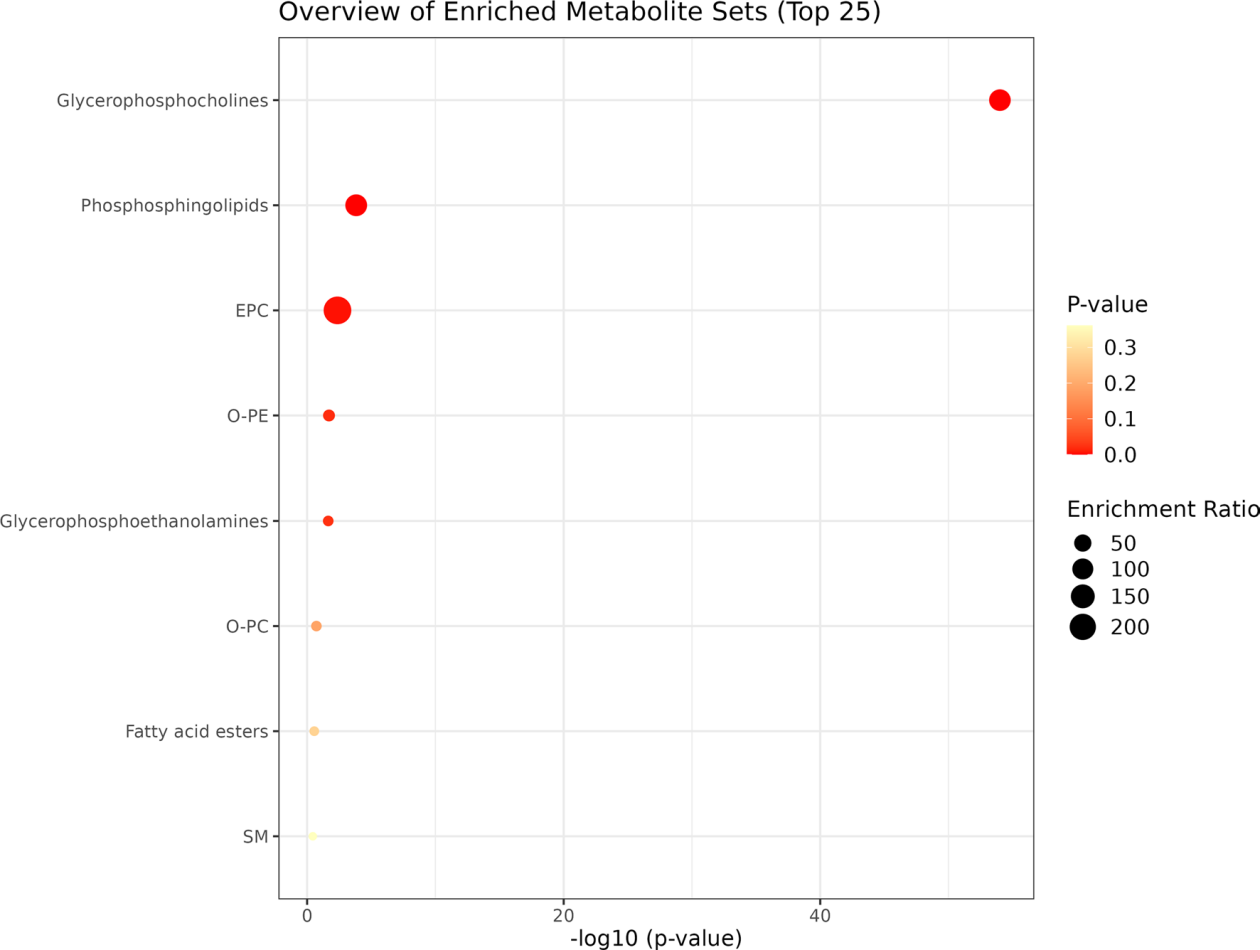
Bubble plot illustrating the over-representation analysis of metabolites associated with the green module from plasma WGCNA analysis

Regarding the functional enrichment of the green module (correlated exclusively with the FP group; Fig. 6), two metabolic sub-classes involved in lipid metabolism (glycerophosphocholines [*p* = 8.7e-52]; and phosphosphingolipids [*p* = 0.066]) were identified.

### Microbiome functional enrichment analysis

The microbiome functional enrichment analysis of each significant module revealed some relevant metabolic pathways (adjusted p-value ≤ 0.1) associated with the prenatal groups.

**Fig. 7:**
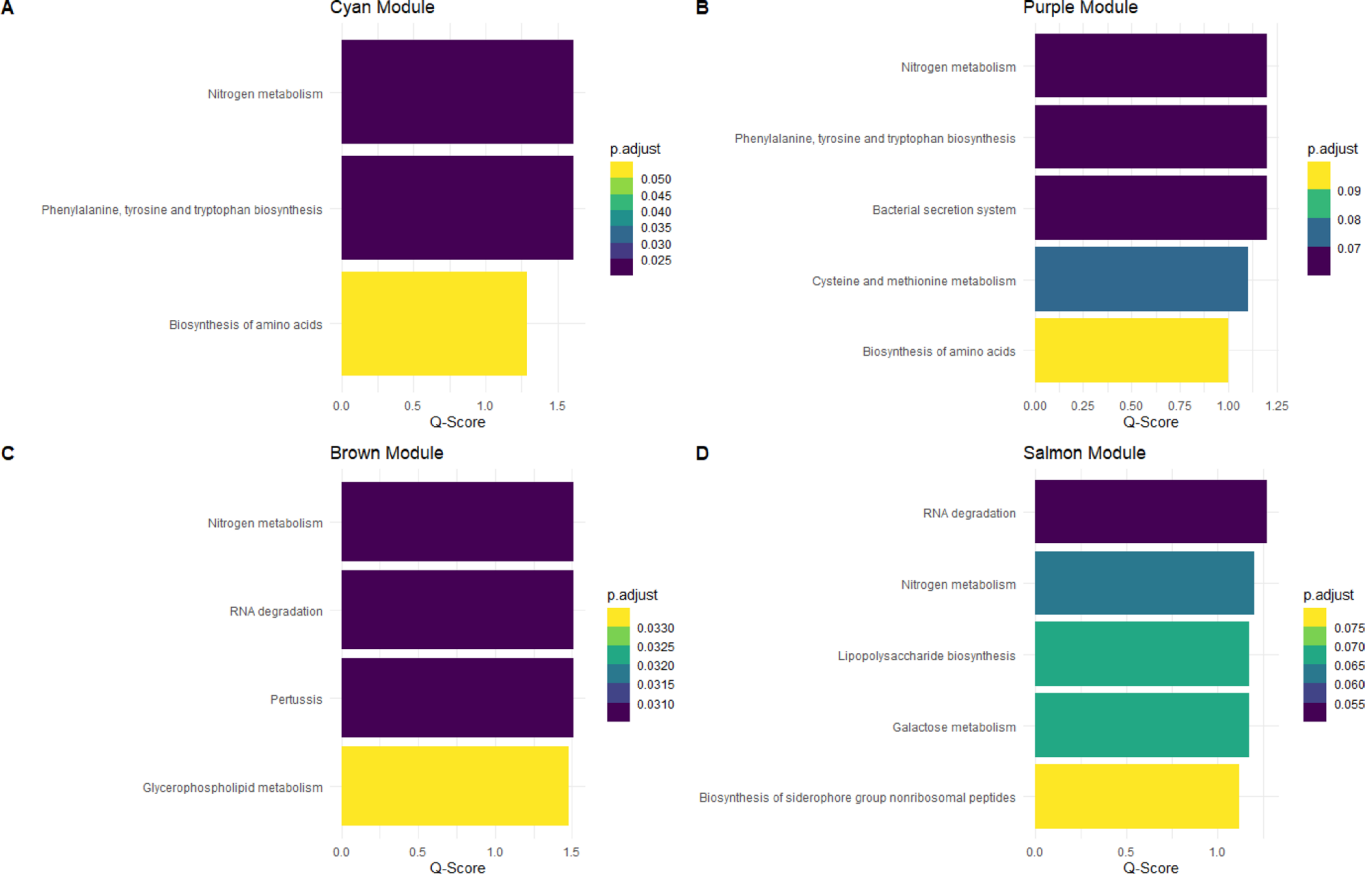
Bar plots illustrating the over-representation analysis of fecal microbiome from the significant modules correlated in the WGCNA. **A** Significant metabolic pathways associated with ASVs in the cyan module. **B** Significant metabolic pathways associated with ASVs in the purple module. **C** Significant metabolic pathways associated with ASVs in the brown module. **D** Significant metabolic pathways associated with ASVs in the salmon module

In the fecal microbiome (Fig. 7), we identified four functionally enriched modules (cyan, purple, brown and salmon). In the cyan module (Fig. 7A), three metabolic pathways were significantly enriched (nitrogen metabolism [*p* = 0.024]; phenylalanine, tyrosine and tryptophan biosynthesis [*p* = 0.024]; and biosynthesis of amino acids [*p* = 0.051]). Regarding the purple module (Fig. 7B), five metabolic pathways were significantly enriched (nitrogen metabolism [*p* = 0.062]; Phenylalanine, tyrosine and tryptophan biosynthesis [*p* = 0.062]; Bacterial secretion system [*p* = 0.062]; cysteine and methionine metabolism [*p* = 0.078]; and biosynthesis of amino acids [*p* = 0.099). The brown module (Fig. 7C) presented four metabolic pathways significantly enriched (nitrogen metabolism [*p* = 0.031]; RNA degradation [*p* = 0.031]; pertussis [*p* = 0.031]; and glycerophospholipid metabolism [*p* = 0.033]). A total of five metabolic pathways were identified associated with the salmon module (Fig. 7D) including RNA degradation [*p* = 0.053], nitrogen metabolism [*p* = 0.062], lipopolysaccharide biosynthesis [*p* = 0.067], Galactose metabolism [*p* = 0.067], and biosynthesis of siderophore group nonribosomal peptides [*p* = 0.075]). No significant metabolic pathways were identified in the pink module.

**Fig. 8:**
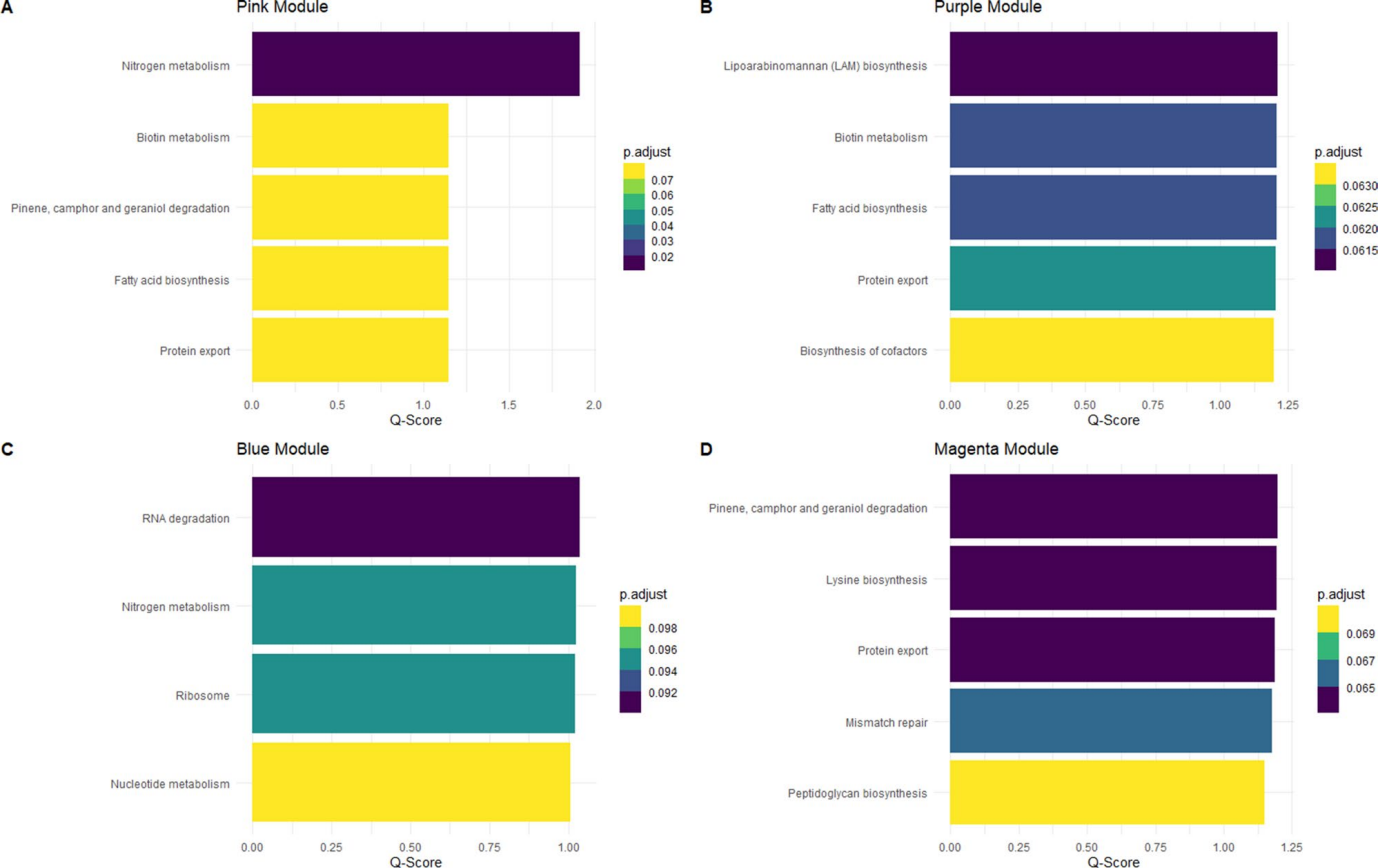
Bar plots illustrating the over-representation analysis of rumen fluid microbiome from the significant modules correlated in the WGCNA. **A** Significant metabolic pathways associated with ASVs in the pink module. **B** Significant metabolic pathways associated with ASVs in the purple module. **C** Significant metabolic pathways associated with ASVs in the blue module. **D** Significant metabolic pathways associated with ASVs in the magenta module

In the rumen fluid microbiome (Fig. 8), we also found four significant enriched modules (pink, purple, blue and magenta). The pink module (Fig. 8A) presented five significant metabolic pathways enriched (nitrogen metabolism [*p* = 0.012]; biotin metabolism [*p* = 0.071]; pinene, camphor and geraniol degradation [*p* = 0.071]; fatty acid biosynthesis [*p* = 0.071]; and protein export [*p* = 0.071]). The purple module (Fig. 8B) showed five significant metabolic pathways enriched (lipoarabinomannan (LAM) biosynthesis [*p* = 0.061]; biotin metabolism [*p* = 0.062]; fatty acid biosynthesis [*p* = 0.062]; protein export [*p* = 0.062]; biosynthesis of cofactors [*p* = 0.063]). In the blue module (Fig. 8C), four metabolic pathways were significantly enriched (RNA degradation [*p* = 0.091]; nitrogen metabolism [*p* = 0.094]; ribosome [*p* = 0.095]; and nucleotide metabolism [*p* = 0.098]. Lastly, the magenta module (Fig. 8D) showed five metabolic pathways enriched (pinene, camphor and geraniol degradation [*p* = 0.063]; lysine biosynthesis [*p* = 0.063]; protein export [*p* = 0.064]; mismatch repair [*p* = 0.066]; and peptidoglycan biosynthesis [*p* = 0.071].

### Metagenomics-metabolomics correlation analysis

**Fig. 9:**
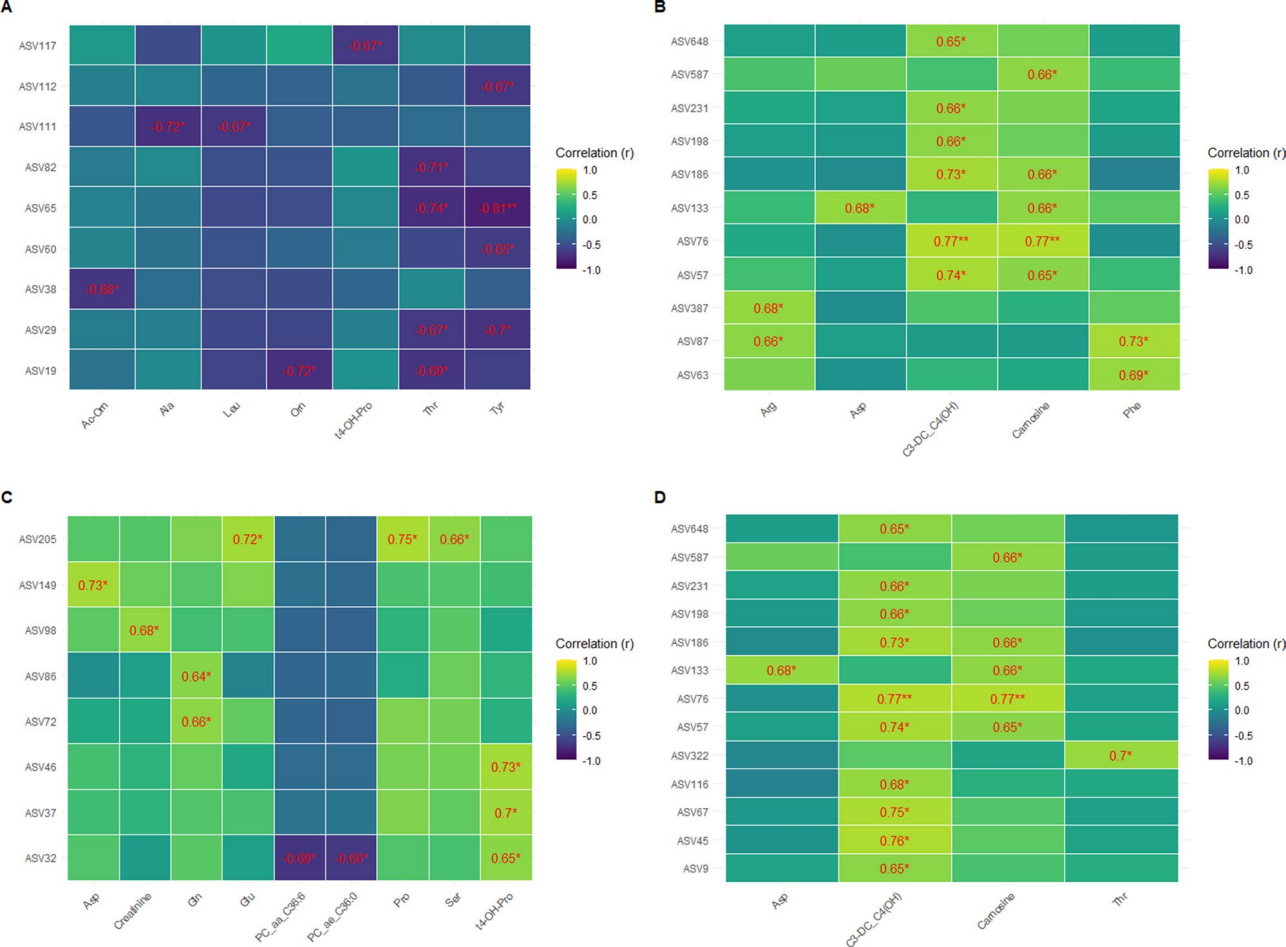
Heatmaps demonstrating the significant correlations between the host plasma metabolome and its fecal and rumen fluid microbiome. **A** NP group correlation between its significant metabolites and its significant fecal ASVs. **B** NP group correlation between its significant metabolites and its significant rumen fluid ASVs. **C** FP group correlation between its significant metabolites and its significant fecal ASVs. **D** FP group correlation between its significant metabolites and its significant rumen fluid ASVs. The heatmaps were reduced to just display components with at least one significant correlation. Significant correlations are numerically represented in the heatmap, and the significant levels are demonstrated according to the p-values threshold (*p-value < 0.01; **p-value < 0.001)

Figure 9 shows the significant correlations observed based on the Spearman’s correlation analysis between significant plasma metabolites and ASVs for each treatment (NP and FP).

In the NP treatment, we identified 13 significant correlations between fecal ASVs and plasma metabolites (Fig. 9A). The correlation coefficients ranged from|0.65| to|0.81|, with threonine and tyrosine accounting for eight of these significant correlations. Tyrosine exhibited the strongest correlation (*r* = -0.81) with ASV65 (*Romboutsia* genus), followed by threonine, which showed the second highest correlation with the same ASV (*r* = -0.74).

The correlations between rumen fluid ASVs and plasma metabolites in the NP treatment (Fig. 9B) showed 16 significant correlations ranging from|0.65| to|0.77|. Two metabolites, malonylcarnitine (C3-DC C4(OH)) and carnosine, accounted for 11 out of the 16 significant correlations identified. The top two correlations were associated with malonylcarnitine and ASV76 (*Saccharofermentans* genus; *r* = 0.77), and carnosine and the same ASV (*r* = 0.77).

Regarding the FP treatment, the correlation between plasma metabolites and fecal microbial taxa revealed 12 significant correlations ranging from|0.64| to|0.75| (Fig. 9C). This assessment did not show significant correlations concentrated in specific metabolites, unlike previous findings. The top two highest correlation values were between proline and ASV205 (*Agathobacter* genus; *r* = 0.75), and between aspartate and ASV149 (*Clostridium sensu stricto 1* genus; *r* = 0.73).

The correlations between rumen fluid microbial taxa and plasma metabolites in the FP treatment (Fig. 9D) showed 17 significant correlations ranging from|0.65| to|0.77|. Most of the correlations (15 out of 17), including the highest ones, were associated mainly with malonylcarnitine and carnosine. Malonylcarnitine is involved in five of the top six correlations. These include its correlation with *Saccharofermentans* genus (ASV76 and ASV186; *r* = 0.77 and *r* = 0.73, respectively), *Acetitomaculum* genus (ASV45 and ASV67; *r* = 0.76 and *r* = 0.75, respectively) and *Christensenellaceae R-7 group* genus (ASV57; *r* = 0.74).

### Metagenome-metabolome metabolic pathways analysis

We refined the first findings (32 significant pathways for the NP group and 26 significant pathways for the FP group) by focusing on the distinct metabolic pathways related with each nutritional treatment group (NP and FP). A total of 10 unique metabolic pathways were found for NP treatment including methane metabolism [FDR = 0.029], pantothenate and CoA biosynthesis [0.037], thiamine metabolism [FDR = 0.029], carbapenem biosynthesis [FDR = 0.039], novobiocin biosynthesis [FDR = 0.039], phenylalanine, tyrosine and tryptophan biosynthesis [FDR = 0.043], staurosporine biosynthesis [FDR = 0.073], phenylalanine metabolism [FDR = 0.076], porphyrin metabolism [FDR = 0.088] and phenylpropanoid biosynthesis [FDR = 0.096]), which are involved with processes such as amino acid and methane metabolism, as well as antibiotics biosynthesis.

**Fig. 10:**
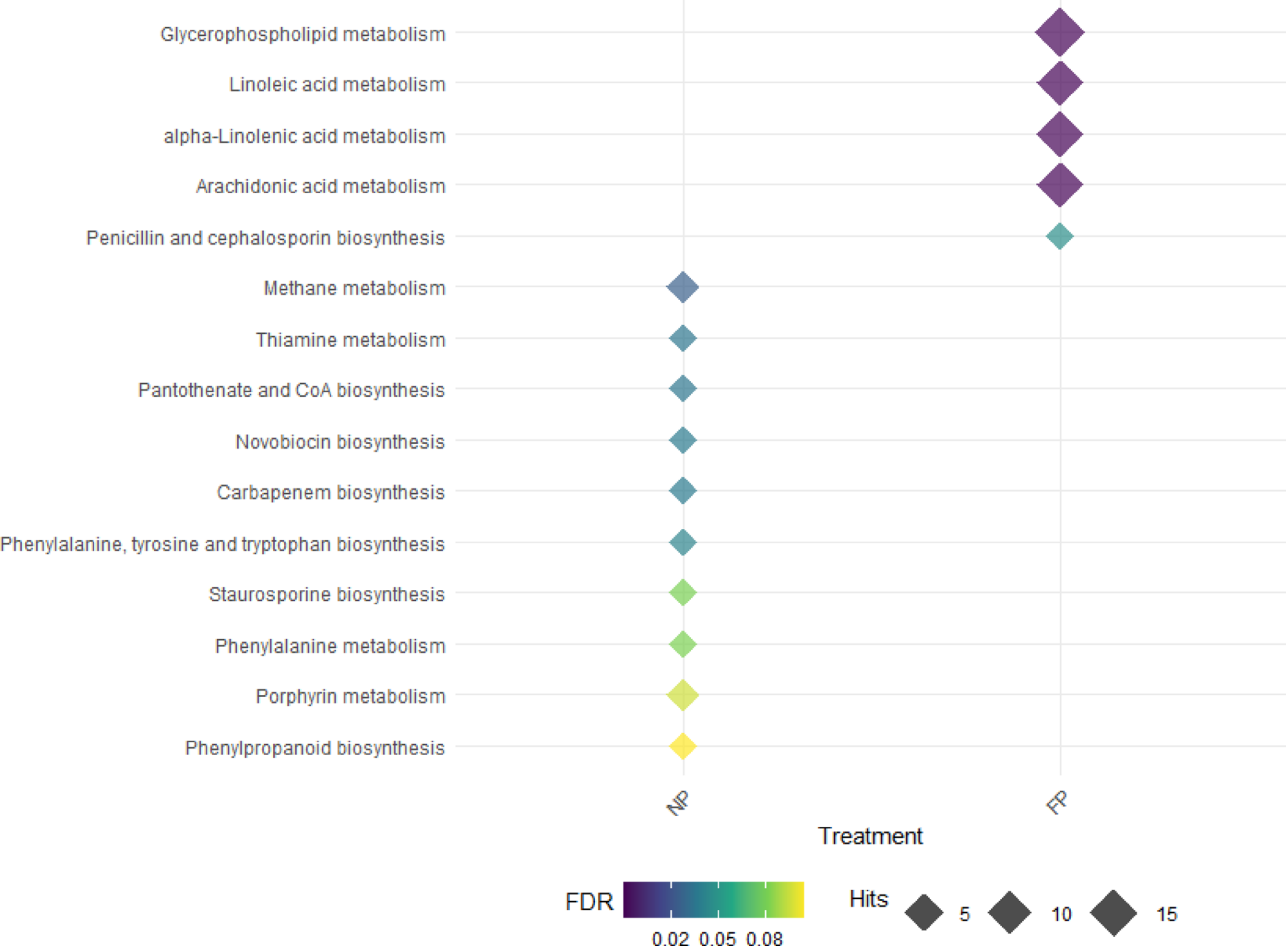
Diamond plot showing the holistic integration of significant metabolites and ASVs (fecal and rumen fluid) in each prenatal nutrition group (NP and FP). The figure illustrates all the significant exclusive metabolic pathways associated with each group

In the FP group, five unique metabolic pathways were identified, predominantly related to lipid metabolism (i.e., glycerophospholipid metabolism [FDR = 8.24e-25], linoleic acid metabolism [4.0e-25], alpha-linolenic acid metabolism [FDR = 3.44e-17], arachidonic acid metabolism [FDR = 8.92e-14]), and antibiotic biosynthesis (Penicillin and cephalosporin biosynthesis [FDR = 0.047]). Figure 10 shows the unique metabolic pathways underlying each prenatal nutritional approach.

## Discussion

Prenatal nutrition can affect fetal growth and development through epigenetic alterations, which may consequently impact the short and long-term metabolism of the offspring [44–47]. Alongside this, the -OMICs integration studies are becoming increasingly essential for improving knowledge about the functional and biological processes in all organisms [48].

### Prenatal nutrition effects on plasma metabolic pathways

From the two significant co-abundance modules of plasma metabolome, we identified several pathways impacted by prenatal nutrition. The yellow module showed that all 13 significant metabolic pathways were highly associated with amino acid metabolic processes. Although the yellow module showed significant correlation with both NP and FP groups, the correlation coefficients were inverses, indicating different modulation of these pathways.

Amino acids play important roles, such as energy sources, primary producers of C1 carbon compounds, anaplerotic metabolites providing intermediates for the tricarboxylic acid (TCA) cycle and gluconeogenesis, precursors for numerous hormones, neurotransmitters, and other specialized metabolites [49].

Biosynthesis of amino acids, as well as glycine, serine and threonine metabolism have already shown an association with prenatal nutrition on fetal skeletal muscle in beef cattle [50]. According to Elolimy et al. [51], the prenatal methionine supplementation during late pregnancy affected metabolic pathways including alanine metabolism, phenylalanine and tyrosine metabolism, glutamate metabolism, arginine and proline metabolism, and tryptophan metabolism during the preweaning period of offspring. Although, valine, leucine and isoleucine degradation pathways have been identified by the same authors, our study revealed that prenatal nutrition affected the biosynthesis instead of the degradation of this metabolic pathway. In general, these metabolic pathways are similar with our present findings in the yellow module. Additionally, our previous studies [29, 52] have identified the effect of prenatal nutrition on similar amino acid metabolic pathways as the present study (arginine biosynthesis; histidine metabolism; beta-alanine metabolism). These findings indicate that the metabolic alterations caused by prenatal nutrition persist until the animal’s slaughter.

Arginine and proline play important roles in proper cellular function and development by participating in DNA and RNA synthesis, protein glycosylation, and detoxification [53, 54]. Phenylalanine, tryptophan, and tyrosine act as precursors to neurotransmitters including dopamine and serotonin [55]. The branched-chain amino acids (BCAA) are composed by leucine, isoleucine and valine, playing roles such as protein metabolism, energy homeostasis (lipid and glucose), gut health and immunity [56]. Therefore, we speculate that prenatal nutrition plays a pivotal role in modulating the intricate crosstalk between the blood, brain, and gastrointestinal tract. This modulation has the potential to influence key physiological processes, including nutrient metabolism, neurotransmitter synthesis, and immune responses, thereby shaping developmental and functional outcomes.

Regarding the green module, which was significant exclusively for the FP group, the metabolites were over-represented in the glycerophosphocholine (GPC) and phosphosphingolipid sub-classes. The GPC metabolite subclass, also known as choline alfoscerate, functions as a choline precursor and is closely related to phosphatidylcholine. It plays essential roles in cell membrane integrity, osmoregulation, signaling, and lipid transport [57, 58]. Phosphosphingolipids, an important subclass of lipids within the sphingolipid class, are involved in key biological processes such as plasma membrane composition, cell signaling, proliferation and differentiation, stress responses, apoptosis, insulin resistance, aging, cancer, and lipid signaling pathways [59, 60]. In beef cattle, Menezes et al. [61] reported that maternal nutrition, particularly maternal body weight gain, changed the hepatic fetal lipid composition in the first trimester of pregnancy. These findings suggest that the over-representation of GPC and phosphosphingolipids in the FP group may reflect changes in lipid metabolism and processes related to cell signaling and osmoregulation, which are crucial for energy balance and cellular function during development.

### Prenatal nutrition effects on microbiome metabolic pathways

The cyan and purple fecal microbiome modules were correlated with the NP group (*r* = -0.47 and *r* = 0.49, respectively). Despite showing moderate correlations [62], these modules were biologically relevant because four of the five significant metabolic pathways identified in the NP fecal microbiome were closely related to amino acid metabolism, including nitrogen metabolism, phenylalanine, tyrosine and tryptophan biosynthesis, cysteine and methionine metabolism, and biosynthesis of amino acids. Notably, these pathways are similar to those observed in the NP plasma metabolome. Given the moderate correlations and the strong functional relevance of these pathways, we focused on these modules as they highlight important metabolic processes potentially influenced by prenatal nutrition. The PP group was also correlated with the purple module; however, showing an inverse correlation regarding the NP group. This indicates different modulation of the metabolic pathways involved in the purple module. Furthermore, the NP metabolic levels of tyrosine were negatively correlated with four ASVs (ASV29 [*Paeniclostridium*], ASV60 [*Romboutsia*], ASV65 [*Romboutsia*], ASV112 [*Romboutsia*]). This finding, alongside the tyrosine biosynthesis pathway identified, highlights the strong association among *Romboutsia* genera, tyrosine and NP group.

The gut microbiota is essential for amino acid production, particularly de novo biosynthesis. Several in vitro studies have revealed that ruminal bacterial species engage in the de novo biosynthesis of amino acids when physiological peptide amounts are present [63]. Furthermore, the gut microbiota profile is determinant for the tryptophan catabolite levels in the systemic circulation [60, 61]. These findings are consistent with our plasma metabolic pathway results, which highlight the interplay between prenatal nutrition, host metabolism, and the gut microbiome. Our correlation results align with these findings, as the NP fecal microbiome demonstrated significant associations with metabolites, five of which belong to the amino acid class. While this suggests a potential link between the NP group and amino acid metabolism, further investigation is required to establish causation or fully substantiate this connection.

The FP group was exclusively correlated with the brown (*r* = -0.59) and salmon (*r* = -0.56) modules. While nitrogen metabolism was also over-represented in the FP group, most of the important metabolic pathways found were not related to amino acid metabolism. Pathways including RNA degradation, glycerophospholipid metabolism, and lipopolysaccharide (LPS) biosynthesis were identified. This is an intriguing discovery, given that GPC and phosphosphingolipid were over-represented in the FP plasma metabolome. Nevertheless, the correlation results demonstrated that only two metabolites from the phospholipid class (PC aa C36:6 and PC ae C36:0) were significantly correlated with a fecal microbial taxon (ASV32 [*Faecalibacterium*]).

Although our study did not specifically investigate the epigenome or related molecules, the RNA degradation pathway identified in the FP group is associated with these factors; thus, we discuss their potential relevance in this context. Epigenetic alterations and regulatory mechanisms, like RNA degradation, represent main processes via which gut bacteria can affect the host health [64, 65]. Cortese et al. [66] has demonstrated that bacteria may directly cause epigenetic alterations in the host, as indicated by distinct patterns of DNA modification in immature intestinal epithelial cells after exposure to commensal or pathogenic pathogens. Notably, the same study discovered that prenatal glucocorticoid-induced epigenetic programming leads to changes in gut microbiota composition in mice, demonstrating intricate connections between the microbiome and epigenome. In addition, alterations in the LPS biosynthetic pathways have been reported in cesarean delivered neonates in comparison with vaginally delivered neonates [67]. The LPS is a surface membrane component of gram-negative bacteria and is recognized by toll-like receptor 4 (TLR4) on the membranes of intestinal epithelial cells, which stimulates components of the immune system [67]. The epigenetic alterations caused by prenatal nutrition may be reflected in the long-term gut microbiome, potentially leading to immunomodulatory effects. In summary, considering the prenatal nutrition effects on fecal microbiome pathways, we can clearly identify a close association among the prenatal nutrition group, the over-represented pathways in the fecal microbiome, and those in the plasma (discussed in the Sect. “Prenatal nutrition effects on plasma metabolic pathways”).

Regarding the ruminal fluid microbiome, the metabolic pathways detected in the NP group consisted mainly of protein metabolism (nitrogen metabolism, protein export), followed by vitamin metabolism (biotin metabolism), and lipid metabolism (fatty acid biosynthesis). Biotin (vitamin B7) is a water-soluble vitamin that plays a significant role in mammal health and illness as a coenzyme for carboxylases involved in a variety of metabolic pathways, including the cellular stress response, gene regulation, and immunological responses [68–70]. In the rumen, the metabolism of proteins and fatty acids is closely linked to microbial activity and fermentation processes. The anaerobic nature of the rumen involves partial breakdown of substrates (including proteins), and the end fermentation products involve volatile fatty acids (VFAs; mainly acetate, propionate, and butyrate), in addition to CO_2_ [71]. As ruminants consume dietary protein, it is quickly digested in the rumen into peptides and amino acids, resulting in ammonia generation and nitrogen loss. When nitrogen retention is inefficient, financial expenses increase due to the demand for more dietary protein. In severe cases, high amounts of rumen ammonia can induce metabolic stress in the animal, whereas excessive nitrogen excretion in manure might harm the ecosystem [72]. Despite the fact that three of the five significant metabolites in the NP rumen fluid ASVs correlation analysis belonged to the amino acid class, malonylcarnitine and carnosine exhibited a higher number and stronger degree of correlations. Given that the correlation analysis of NP fecal ASVs included more than 80% of the significant correlated metabolites belonging to amino acids, these findings may indicate a reduced relationship between the rumen fluid microbiota and amino acid metabolites. The FP rumen fluid correlations were similar to NP rumen fluid correlations, with malonylcarnitine and carnosine demonstrating several significant correlations with different ASVs. More research combining host metabolites and microbiota is needed to fully understand these relationships.

The PP group, in addition to the previously mentioned pathways, also exhibited regulatory mechanisms related to RNA degradation and nucleotide metabolism, while the FP group was associated with processes like mismatch repair. These findings suggest potential regulatory roles of prenatal nutrition in microbiome-mediated pathways. However, further research is needed to address the gaps in the literature regarding the complex interactions between epigenetic modifications, regulatory mechanisms, and the microbiome, particularly in the context of prenatal nutrition and its long-term effects on offspring development. In summary, our results reveal significant divergences in the ruminal and fecal microbiomes of offspring from different prenatal nutritional treatments despite uniform management throughout their productive cycle. These microbial differences are interconnected with the host metabolome, influencing multiple metabolic pathways including protein, lipid, and vitamin metabolism, as well as regulatory mechanisms in the ruminal fluid microbiome. This interconnection underscores the lasting impact of prenatal nutrition on offspring development and metabolic function.

### Metabolomics-metagenomics integration approach reveals lipid and methane metabolism as main pathways impacted by prenatal nutrition

The results found in the holistic integration analysis revealed the most important metabolic pathways involved in the long-term impacts of prenatal nutrition in beef cattle offspring. The NP group demonstrated close associations with amino acid metabolism and methane metabolism, while the FP group showed a strong relationship with lipid metabolism.

Regarding the NP group, we found some pathways similar with our previous analyses (phenylalanine, tyrosine and tryptophan biosynthesis; phenylalanine metabolism) related to amino acid metabolic processes. However, the most significant metabolic pathway identified was associated with methane metabolism. Ruminants emit 16% of worldwide methane (CH_4_), with the beef cattle and dairy sectors accounting for 35% and 30%, respectively [73]. Methane is an unnecessary by-product of microbial fermentation of mostly complex carbohydrates in the rumen, produced by methanogenic archaea and expelled into the environment via the animal’s mouth and nose. Eructated methane from ruminal microbial fermentation not only contributes to global warming but also wastes energy, reducing feed efficiency [74]. Given the microbiome’s crucial role in methane production, strategies to mitigate emissions in ruminants primarily target dietary interventions that alter rumen conditions and microbial ecology [75–77]. Our findings suggest a novel association between prenatal nutrition and methane metabolism pathways in the offspring’s ruminal microbiome. This association could have implications for feed efficiency, as previous research has shown that less feed-efficient steers have a higher relative abundance of methanogens in their ruminal microbial populations compared to high feed-efficient steers [78]. Our results raise the intriguing possibility that prenatal nutrition throughout gestation might influence the animal’s long-term feed efficiency and methane production capacity. Further research is needed to elucidate the mechanisms underlying this association and its potential applications in sustainable livestock management and methane mitigation strategies [81].

Interestingly, the FP group continues to show a strong association with lipid metabolism, as demonstrated in our previous analyses performed in this study. Glycerophospholipid metabolism (also identified in the brown module of the fecal microbiome), as well as linoleic acid, alpha-linolenic acid, and arachidonic acid metabolism pathways, were shown as the main pathways affected by the FP group in a holistic perspective. Lipid metabolism is a pivotal focus in livestock industry research, whether for milk or meat production [79]. In addition to the lipid roles already discussed (available in the discussion Sect. 4.1 and 4.2), the PUFAs (polyunsaturated fatty acids) play several other important roles. The intramuscular fat content in beef typically includes only 5% PUFAs [80], which can vary depending on nutritional factors [81]. A high ratio of omega-6 (n-6; such as linoleic acid and arachidonic acid) in relation to n-3 fatty acids promotes several illnesses, including cardiovascular disease, arthritis, and cancer, whereas lower levels have suppressive effects in humans [82], pigs [83–85] and mice [86]. Association between maternal low-protein diet and disordered regulation of lipid metabolism in rats was reported [87]. In ruminants, some studies have identified prenatal nutrition effects on lipid metabolism [88, 89]. Supporting our findings, Menezes et al. [61] demonstrated that maternal nutrition significantly influenced fetal hepatic lipid composition in cattle during early gestation. Their study revealed that appropriate maternal nutrition leading to moderate weight gain optimized fetal lipid profiles, resulting in higher omega-3 and lower omega-6 fatty acid abundances in fetal livers. This aligns with our observed alterations in PUFA metabolism pathways. Although Menezes et al. [61] focused on fetal liver tissue, in contrast to our analysis of blood metabolome and fecal microbiome, both studies highlight the broad impact of prenatal nutrition on offspring lipid metabolism. This concordance across different experimental approaches suggests that prenatal nutrition may have lasting effects on various aspects of lipid metabolism in offspring.

In general, our findings corroborate to novel discoveries, particularly related to the association among prenatal nutrition, methane metabolism and PUFA metabolism in beef cattle. Further studies are needed to assess more in-depth the linkage between beef prenatal nutrition and these pathways.

## Conclusions

Prenatal nutrition modulated several metabolic pathways in bulls, mainly involved with protein, lipid and methane metabolism, as well as some regulatory mechanisms. Based on all the analyses performed, we conclude that the NP group had a high association with amino acid metabolic processes and methane metabolism, while the FP group showed a strong relationship with lipid metabolism, mainly PUFA metabolism. The PP group demonstrated metabolic pathways associated with regulatory mechanisms and amino acid metabolism. It is important to note that, in addition to exclusive modules within each treatment, modules shared between groups revealed in all contrasts opposing correlations, presumably indicating epigenetic modifications caused by prenatal nutrition. Briefly, the integration of plasma host metabolome and metagenomics (fecal and ruminal fluid) has provided intriguing insights into prenatal nutrition in beef cattle, revealing novel discoveries.

## Electronic supplementary material

Below is the link to the electronic supplementary material.

Supplementary Material 1

Supplementary Material 2

Supplementary Material 3

Supplementary Material 4

Supplementary Material 5

Supplementary Material 6

## Data Availability

The metabolome dataset analysed during the current study are available in the supplementary material (Additional file 1), as well as the microbiome datasets (fecal microbiome [Additional file 2]; rumen fluid microbiome [Additional file 3]). The corresponding taxonomy annotation can be found in Additional file 4 (fecal microbiome) and Additional file 5 (rumen fluid microbiome). The hierarchical clustering of WGCNA modules for the -OMIC datasets is shown in Additional file 6.

## Abbreviations

ASVs: Amplicon sequence variants
BCAA: Branched-chain amino acids
BW: Body weight
C3-DC C4(OH): Malonylcarnitine
CH_4_: Methane
CP: Crude protein
DHA: Docosahexaenoic acid
DMI: Dry matter intake
DPA: Docosapentaenoic acid
EPA: Eicosapentaenoic acid
FDR: False discovery rate
FIA-MS/MS: Flow injection analysis-tandem mass spectrometry
FP: Full programming
GPC: Glycerophosphocholine
HPLC: High-performance liquid chromatography
HPLC-MS/MS: Liquid chromatography tandem-mass spectrometry
KEGG: Kyoto encyclopedia of genes and genomes
KO: KEGG ortholog
LAM: Lipoarabinomannan
LOD: Limits of detection
LPS: Lipopolysaccharide
MAPA: Ministry of Agriculture, Livestock, and Supply of Brazil
NP: Not programmed
PP: Partial programming
PUFAs: Polyunsaturated fatty acids
r: correlation
TCA: Tricarboxylic acid
TDN: Total digestible nutrients
TLR4: Toll-like receptor 4
VFAs: Volatile fatty acids
WGCNA: Weighted gene co-expression network analysis
