# Supplementary material for: Impacts of prenatal nutrition on metabolic pathways in beef cattle: an integrative approach using metabolomics and metagenomics
Source: BMC Genomics. 2025 Apr 10;26:359. doi: 10.1186/s12864-025-11545-6 (PMC11983759; doi:10.1186/s12864-025-11545-6)

Additional file 6A. Hierarchical clustering of WGCNA plasma metabolites modules.

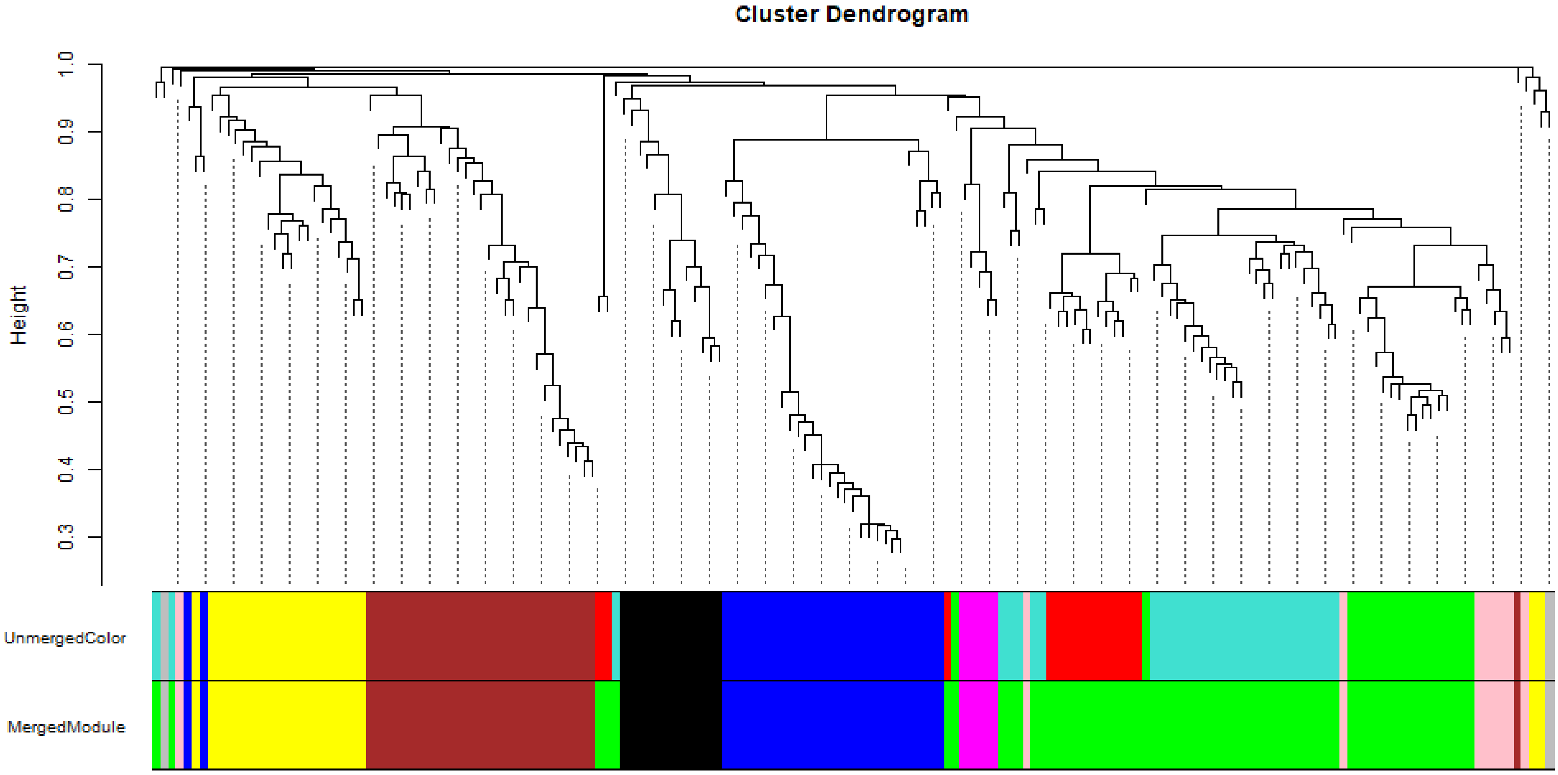

Additional file 6B. Hierarchical clustering of WGCNA fecal ASVs modules.

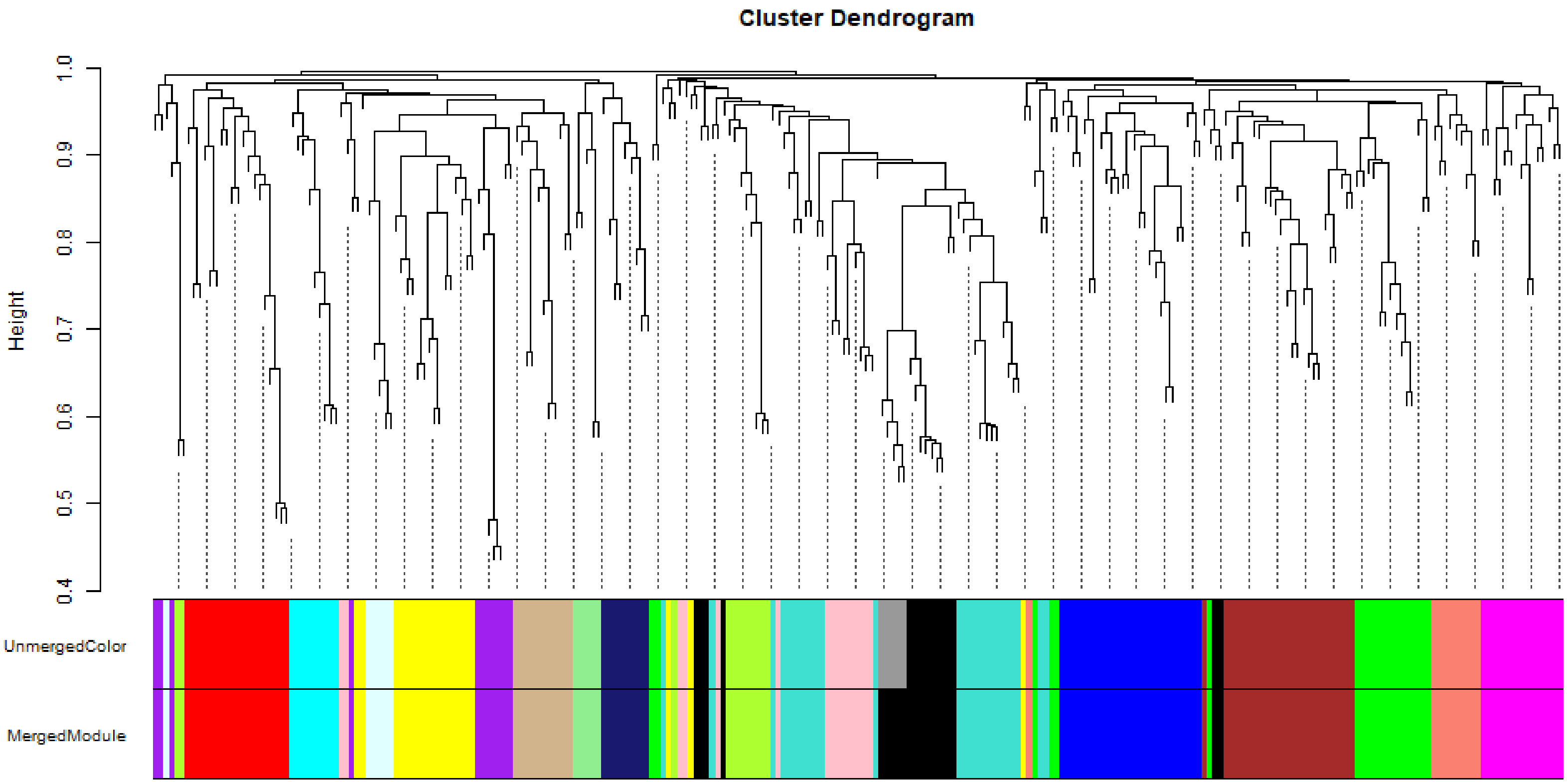

Additional file 6C. Hierarchical clustering of WGCNA rumen fluid ASVs modules.

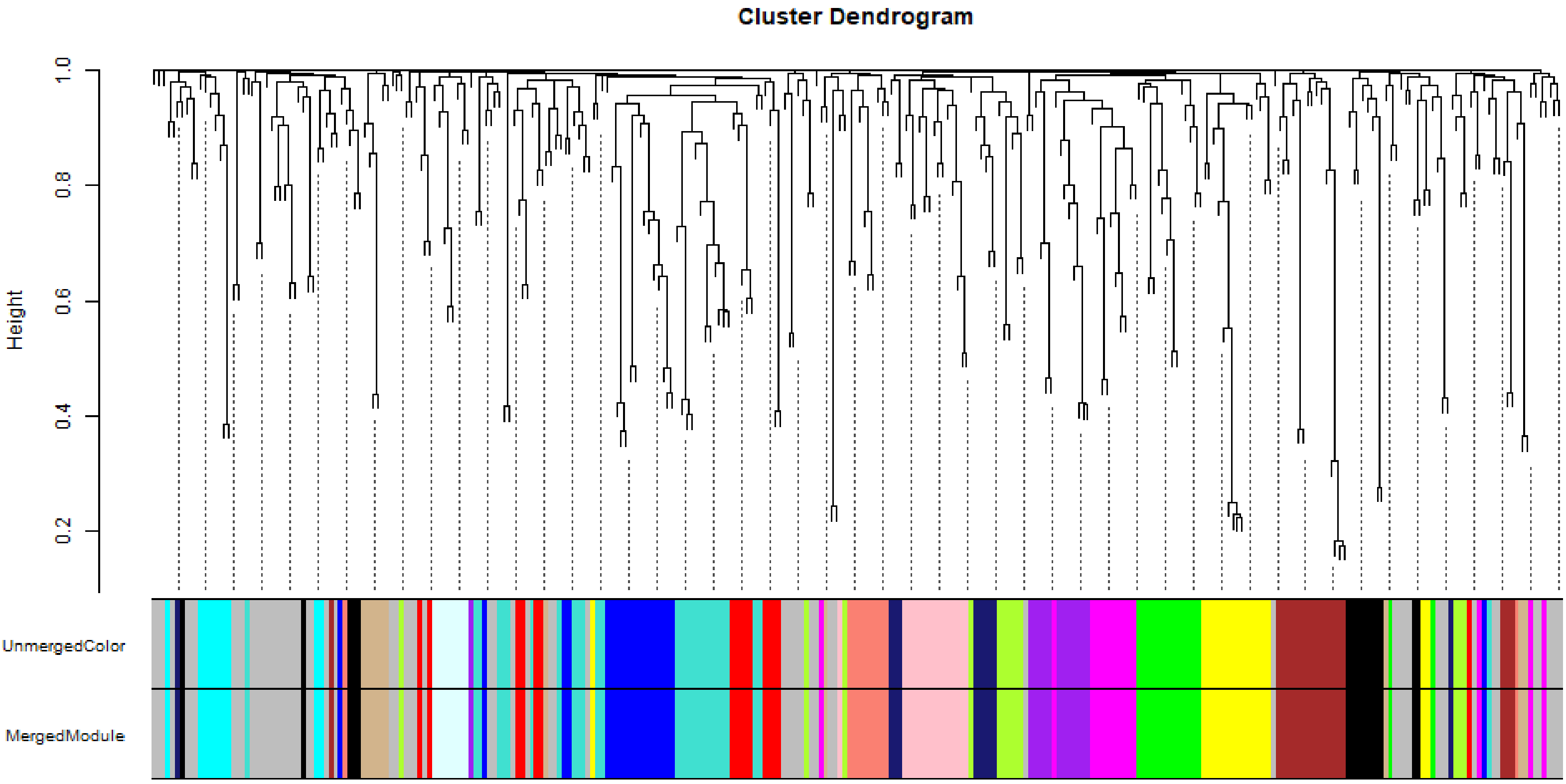

Supplement: Supplementary file 6 — Supplementary Material 6 [file 12864_2025_11545_MOESM6_ESM.pdf]
